# Engineering nanoparticle features to tune Rayleigh scattering in nanoparticles-doped optical fibers

**DOI:** 10.1038/s41598-021-88572-2

**Published:** 2021-04-27

**Authors:** Victor Fuertes, Nicolas Grégoire, Philippe Labranche, Stéphane Gagnon, Ruohui Wang, Yannick Ledemi, Sophie LaRochelle, Younès Messaddeq

**Affiliations:** Centre D’optique, Photonique Et Laser, Université Laval, 2375 Rue de la Terrasse, Québec, QC G1 V 0A6 Canada

**Keywords:** Materials for optics, Nanoscale materials

## Abstract

Rayleigh scattering enhanced nanoparticles-doped optical fibers are highly promising for distributed sensing applications, however, the high optical losses induced by that scattering enhancement restrict considerably their sensing distance to few meters. Fabrication of long-range distributed optical fiber sensors based on this technology remains a major challenge in optical fiber community. In this work, it is reported the fabrication of low-loss Ca-based nanoparticles doped silica fibers with tunable Rayleigh scattering for long-range distributed sensing. This is enabled by tailoring nanoparticle features such as particle distribution size, morphology and density in the core of optical fibers through preform and fiber fabrication process. Consequently, fibers with tunable enhanced backscattering in the range 25.9–44.9 dB, with respect to a SMF-28 fiber, are attained along with the lowest two-way optical losses, 0.1–8.7 dB/m, reported so far for Rayleigh scattering enhanced nanoparticles-doped optical fibers. Therefore, the suitability of Ca-based nanoparticles-doped optical fibers for distributed sensing over longer distances, from 5 m to more than 200 m, becomes possible. This study opens a new path for future works in the field of distributed sensing, since these findings may be applied to other nanoparticles-doped optical fibers, allowing the tailoring of nanoparticle properties, which broadens future potential applications of this technology.

## Introduction

Optical fiber sensors (OFS) technology has been widely explored in last decades and is currently well established in different fields of application, being one of the most active research areas in optical sensing^1^. Their great impact in technological sector is motivated by the several advantages that present silica optical fibers compared to other traditional sensing methods such as their light weight, immunity to electromagnetic interference, resistance to high temperature and harsh environments, possibility to be embedded in different materials and forms or the possibility of hosting multiple sensors in a single fiber, among others^2^. The unique property of providing spatial resolution along the whole fiber under test, while allowing the sensing of different parameters (strain, temperature, 3D shape, refractive index, etc.) makes distributed optical fiber sensors (DOFS) of particular interest from a point of view of applications^3–6^. This kind of sensor exploits the intrinsic scattering along the fiber, mainly Raman, Brillouin and Rayleigh scatterings, for the smart sensing of the different physical parameters under study. Optical time-domain reflectometry (OTDR) based on Rayleigh, Raman, and Brillouin scatterings constituted the first generation of optical fiber sensors, being able of resolving temperature and strain with a spatial resolution from meters up to ~ 10 cm over some kilometers^3,7^. The desired spatial resolution and measurement range determine the scattering mechanism to be selected^5,8^. In the case of OTDR based on Rayleigh scattering, the sensing of temperature and strain has been achieved in long lengths of fiber, ~ km, but with low spatial and temperature resolution, ~ m and ~ 10 °C, respectively^9^. With the aim of improving the spatial resolution of DOFS, the current sensing systems have moved from time-domain to frequency domain. Rayleigh scattering based Optical frequency-domain reflectometry (OFDR) is characterized by higher sensitivity and a spatial resolution of sub-millimeter scale, being suitable for short sensing lengths, < 100 m^9,10^. Currently, optical backscatter reflectometry (OBR) is one of the most popular and relevant of the existing OFDR methods, in which the light reflected back to the detector corresponds to the fiber Rayleigh scattering existing. Therefore, through its analysis, spectral shifts in the Rayleigh backscattering in the core of the optical fiber can be measured, allowing the measurement of changes in strain or temperature^11,12^.

Historically, efforts have been focused on minimizing Rayleigh scattering in optical fibers since it is one of the main sources of losses that contribute to the fiber attenuation. However, because of the promising features of OBR systems, the current trend in DOFS is to engineer optical fibers to enhance the Rayleigh scattering in a controlled way, so as not to increase optical losses disproportionally. Different approaches have been followed to create these enhanced backscattering fibers such as designing high-numerical aperture (NA) fibers (highly Ge- and Ge/B-doped)^9,13^, isotopes irradiation in Ge/P co-doped fibers^14^, increasing scattering cross section UV exposure of a single-mode fiber^9,13,15^, inscription of nanogratings by a femtosecond laser^16^ or the incorporation of nanoparticles as scattering centers into the core of the fiber etc^17–22^. Among them, the most promising and best reported results so far correspond to the last-mentioned approach, proposed by Blanc et al. The authors demonstrate the fabrication of co-doped erbium and MgO-based nanoparticles doped fibers from preforms fabricated by the conventional modified chemical vapor deposition (MCVD) method, in which the growth in situ of a random distribution of MgO-based nanoparticles, is attained thanks to the immiscibility of alkaline-earth ions in silicate systems in the range of temperatures reached during the process^23^. This approach shows several advantages since it allows obtaining nanoparticles-doped fibers that can be handled as a standard fiber, that is, drawn, spooled, stripped, cleaved and spliced as a standard single mode fiber (SMF) while preserving their protective jacket which is essential for some applications^12^. The authors reported several MgO-based nanoparticles fibers, in which the sizes of nanoparticles strongly determine the induced elastic scattering and optical losses. Fibers with MgO-based nanoparticles of 20–160 nm presented an enhancement of the backscattering of 46.1–47.5 dB with respect to a standard SMF-28 fiber but with high optical losses 292–298 dB/m^17,18^, while fibers with nanoparticles < 100 nm of diameter the backscattering enhancement was 32.3–45.2 dB with losses of 27.8–33.1 dB/m^19–22^. Very recently, MgO-based nanoparticles doped fibers have been even more optimized, allowing an increase of the Rayleigh scattering up to 48.9 dB with a two-way attenuation of 14.3 dB/m^24^. Based on these fibers, DOFS has been fabricated for the sensing of different parameters such as refractive index^17,18,22^, strain^20^, temperature^20,21,25^, 3D shape sensing^19^. All of these works, based on MgO nanoparticles-doped fibers have demonstrated that the use of nanoparticles is suitable to fabricate new DOFS for future potential applications. However, the optical losses of these systems, induced by that enhancement of scattering, are still high which restrict considerably their sensing distance.

Therefore, the incorporation of nanoparticles in the core of silica-based fibers for DOFS applications is still in an early stage and there is a need of further studies, especially from a material science point of view, since the behavior of the nanoparticles in the core and their interaction with silica during the high temperatures in the fabrication steps of preform and fiber has been hardly studied. Thus, the optimization of the fabrication processes as well as the investigation of new nanoparticles doped fibers in order to attain DOFS with a better trade-off between scattering enhancement and optical losses, is required for potential future applications. It has been recently demonstrated that drawing conditions strongly affect to the nanoparticle size and distribution inside the core of silica-based optical fibers^26,27^. These findings may be seized to tailor them and consequently the induced light scattering in the optical fibers. However, according to our best knowledge no work, so far, has pursued this target and carried out a detailed study about the influence of drawing conditions in the backscattering enhancement and its correlation with the nanoparticles features.

In this context, the aim of this work is to design novel nanoparticles-doped fibers with tunable enhancement of Rayleigh scattering and optical losses, through the engineering of nanoparticle characteristics by preform and fiber fabrication conditions, for their application as DOFS. For that purpose, Ca-based nanoparticles are grown in situ inside of the core of preforms fabricated by MCVD and the impact of the experimental conditions on the nanoparticle features and the induced light scattering is thoroughly discussed. In order to analyze their potential for distributed sensing, measurements of backscattering enhancement by OBR and microstructural observations of the core by Scanning electron microscopy (SEM) of both the preforms and the optical fibers are included in this study. Attenuation measurements by means of cutback method are also carried out, in order to explore the behavior of Ca-based nanoparticles-doped optical fibers beyond the L-band.

## Materials and methods

### Preform and fiber fabrication

Ca-based nanoparticles doped preforms were fabricated by the conventional MCVD method along with solution doping technique. We started with a high purity grade fused quartz tube of 25 mm outside diameter and 19 mm inside diameter. A porous SiO_2_ soot matrix was deposited inside the quartz tube using the MCVD process. With a constant flow of SiCl_4_ (85 sccm) and a mixture of helium and oxygen inside the rotating substrate tube (50 rpm), the temperature of the substrate tube was precisely controlled at 1350 °C all along the deposition process. The heat source for this process was a hydrogen/oxygen burner moving at a constant speed of 35 mm/min. After the porous soot matrix fabrication, the tubes were dismounted from the MCVD lathe. A nitrogen flow as well as a positive pressure atmosphere was used inside of the preforms to avoid any possible contamination. The porous silica layer was soaked with a solution of CaCl_2_ in water. The size and distribution of the nanoparticles in the final preform and the fiber strongly depend on the concentration of the initial solution. Solutions of 0.1 M, 0.01 M and 0.005 M were studied. Afterwards, the doped silica layer was vitrified at 1800–1950 °C and, finally, the tube was collapsed above 2000 °C. Preforms fabricated from solutions with different concentration and vitrification temperature of the silica soot were considered, labeled as preform A-D, according to the different fabrication conditions included in Table 1. Germanium and phosphorus were used in the same amount for all preforms, in order to create a step-index profile that allows the guidance of light, being the refractive index contrast (Δn) measured of 14 × 10 − ^3^, 11 × 10^−3^ and 9 × 10^−3^ for preforms B, C and D, respectively (Table 1 and Fig. S1). Refractive index contrast of preform A could not be evaluated because of the high density of particles presented absorbed the light (see Fig. S2). Thus, the different nanoparticle concentration has an impact on the Δn measured, increasing it as concentration increases. The different nanoparticle concentration also affects the transparency of the preform core (Fig. S2). The diameter of the preforms was 15 mm with a core diameter of 1.2 mm. Owing to the high temperatures reached during MCVD process, the calcium chloride precursor employed is oxidized. Because of the immiscibility gap that exists for CaO and SiO_2_, when CaO is presented in few moles, according to its phase diagram^28^, Ca-rich based nanoparticles were grown in situ inside the core of the preforms due to a spontaneous phase separation process. This principle is considered as a straightforward technique of growing metal oxides (such as Mg, Ca or Sr) rich nanoparticles with spherical shapes in silicate systems^29^.

**Table 1:** Preform fabrication conditions and refractive index contrast for Ca-based nanoparticles doped preforms.

	Solution concentration (M)	Vitrification temperature (°C)	Δn (×10^–3^)
Preform A	0.1	1950	–
Preform B	0.01	1950	14
Preform C	0.01	1800	11
Preform D	0.005	1800	9

The optical fibers were drawn from the fabricated preform D on a drawing tower at different temperatures in the range 1870–2200 °C, while the preform feed and drawing speed were maintained constant at 0.3 mm/min and 5 m/min, respectively. The fibers were designed to match with SMF-28 telecom fibers, having an external diameter of 125 μm and a core diameter of 10 μm, which allows manipulating them as standard ones.

### Microstructural characterization

It was carried out by means of a FEI QUANTA 3D FEG Scanning Electron Microscopy (SEM), with a resolution of 1.5 nm at 30 kV in Secondary Electron (SE) mode. Composition of the Ca-based nanoparticles and the core of preform and fibers were analyzed by means of energy dispersive X-ray (EDX) detector.

### Optical characterization

Refractive index profile of the fabricated preforms was measured by using a Photon Kinetics PK2600 Preform Analyzers. OBR measurements of the enhanced backscattering fibers were carried out by using a commercial Luna OBR 4600, characterized by a sensitivity of − 130 dB and a spatial resolution of 20 μm. For the measurements, the different nanoparticles-doped fibers were spliced to a SMF-28 fiber pigtail ending in a FC/APC connector and connected to the OBR 4600. The fibers were evaluated with a laser input centered at 1550 nm and a wavelength range of 43 nm, sweeping in the telecommunication window and taking 16,384 sensing points per analysis. Attenuation (*α*) was measured by the standard cut-back method.

The attenuation generated by nanoparticles acting as scattering centers, assuming that Rayleigh scattering is the major source of scattering losses, can be expressed by Eq. (1)^23^:

1 $$ \alpha \left( \frac{dB}{m} \right) = 4.34 \cdot \sigma_{Rayleigh} \cdot N \cdot\Gamma $$

where *σ*_*Rayleigh*_ is the Rayleigh scattering cross section, *N* is the density of nanoparticles (m^−3^) and *Γ* is the overlap factor between the field and the core. *σ*_*Rayleigh*_ is expressed by Eq. (2)^30^:

2 $$ \sigma_{Rayleigh} = \frac{{\left( {2\pi } \right)^{5} }}{48} \cdot \frac{{\emptyset^{6} }}{{\lambda^{4} }} \cdot n_{med}^{4} \cdot \left( {\frac{{n_{np}^{2} - n_{med}^{2} }}{{n_{np}^{2} + 2n_{med}^{2} }}} \right)^{2} $$

where *Ø* is the diameter of the particles, n_med_ and n_np_ are the host material and particle refractive indices, respectively and λ is the wavelength of the incident light.

On the other hand, the overlap factor in a uniformly doped single mode core, is related to the core radius (*r*_*co*_) and the mode field radius (*w*) by Eq. (3).

3 $$ {\Gamma } = 1 - e^{{\left( { - 2\frac{{r_{co}^{2} }}{{w^{2} }}} \right)}} $$

For a step-index single-mode fiber, *w* may be estimated from *r*_*co*_ and the *V* number, by means of Marcuse's equation (Eq. 4)^31^:

4 $$ \frac{w}{{r_{co} }} \approx 0.65 + \frac{1.619}{{V^{3/2} }} + \frac{2.879}{{V^{6} }} $$

where the V number is defined as in Eq. (5):

5 $$ V = \frac{2\pi }{\lambda }r_{co} NA = \frac{2\pi }{\lambda }r_{co} \sqrt {n_{core}^{2} - n_{cladding}^{2} } $$

## Results and discussion

### Influence of preform fabrication conditions on nanoparticles features: microstructural characterization

**Figure 1:**
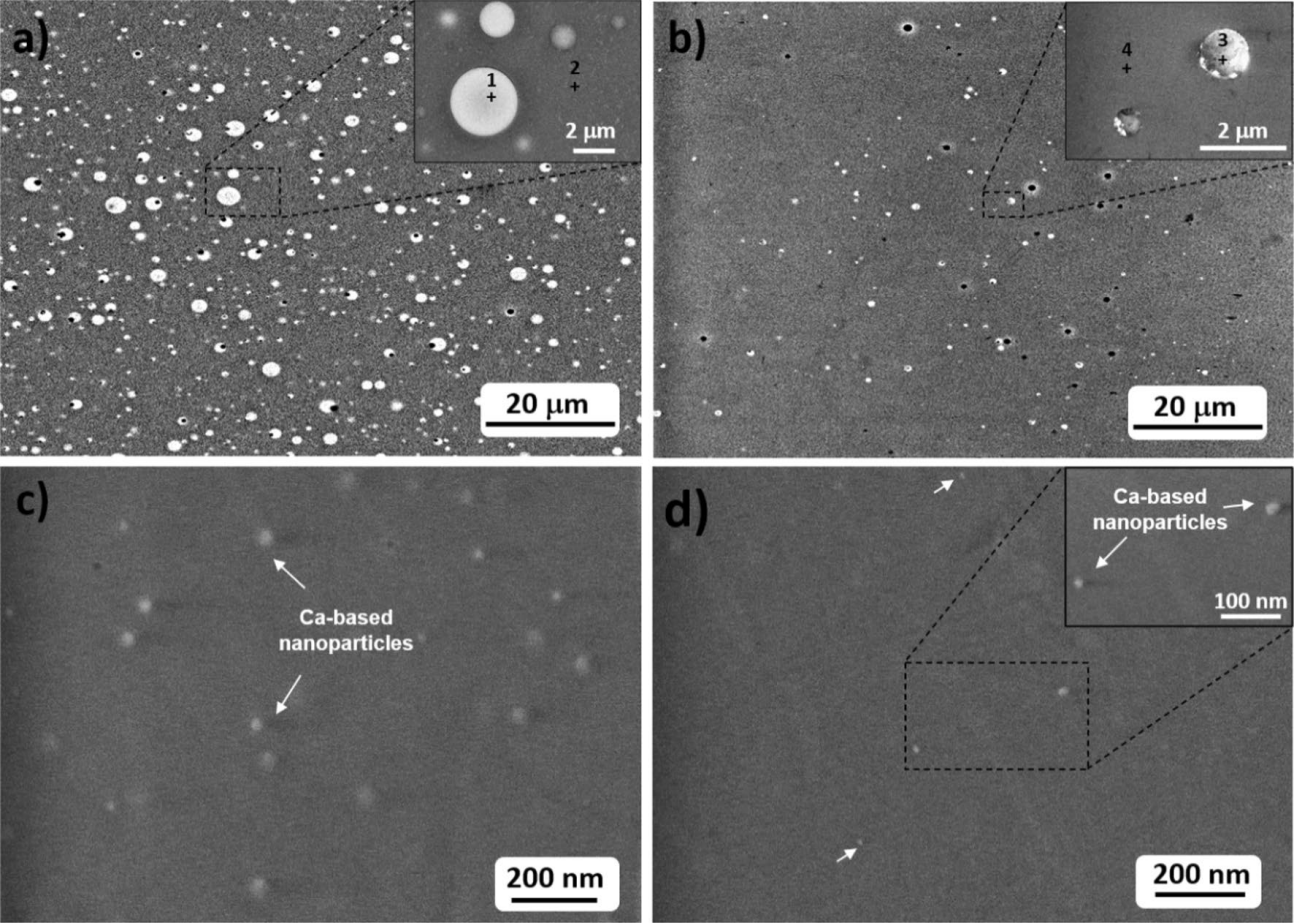
SEM micrographs of the core of: (**a**) preform A, (**b**) preform B, (**c**) preform C, (**d**) preform D. The inset of (**a**) and (**b**) shows an enlargement of the marked area in the corresponding core micrograph, showing in more detail the nanoparticles. EDX analysis was carried out on points 1–4 and the core of preforms C and D and the composition is included in Table 2.

Solutions with different concentrations were considered for solution doping during preform fabrication, from 0.05 to 0.1 M, as well as different temperatures of vitrification of silica soot, 1800 °C and 1950 °C (see "Materials and methods" for more details). Their influence on the size of the nanoparticles and particle distribution in the core was studied by SEM (Fig. 1). Figure 1a shows SEM micrographs of the core of preform A, in which a high density of particles is observed, with sizes ranging from 500 nm to 3.3 μm in diameter. As consequence of the large size of the microparticles presented along the preform core, homogeneously distributed, and their high density, its color is milky (Fig. S2). Figure 1b shows the core of preform B, in which it can be clearly seen that decreasing the concentration from 0.1 to 0.01 M has a direct impact on the size distribution of the particles formed in the core and the density, decreasing both considerably. The smallest particles are of 250 nm in diameter while the largest ones are about 1 μm. Consequently, the core of the preform became more translucent (Fig. S2). The composition of the particles and the glass was investigated by EDX in the two points marked in the inset of Fig. 1a,b. As it can be seen in Table 2, it is demonstrated that particles grown in situ are Ca-enriched (40–45 mol.%), as previous works suggested^28,32^ and therefore the phase separation of CaO and SiO_2_ is occurring during preform fabrication process. From the EDX analysis, it is also inferred that phosphorus tends to be present in the particles, ~ 11 mol.%, in accordance with^33^, while in the rest of the core germanium is more abundant, ~ 4–5 mol.% and calcium is not found. The silicate glass has a silica content of about 94–95 mol.%.

**Table 2:** Oxide composition (mol.%) obtained by EDX analyses on points 1–4 marked in Fig. 1 for Ca-based nanoparticle doped preforms.

	SiO_2_	CaO	P_2_O_5_	GeO_2_
Point 1 (preform A)	40.31	44.93	10.91	3.85
Point 2 (preform A)	94.24	–	0.30	5.46
Point 3 (preform B)	45.79	40.76	10.59	2.86
Point 4 (preform B)	95.15	–	0.28	4.57
Core preform C	92.88	0.65	0.82	5.65
Core preform D	93.42	0.39	0.76	5.42

In preforms C and D, vitrification temperature was decreased from 1950 to 1800 °C and both the density and size of the particles were strongly reduced if compared to preforms A and B. As it is shown in Fig. S3a,b no micrometric particle is observed in SEM micrographs at low magnification, unlike in preform A and B (Fig. 1a,b), which increases considerably the transparency of the core (Fig. S2). In both preforms, isolated nanoparticles were found (Fig. 1c, d), with a size distribution and density smaller for preform D than C, as expected. This fact is also reflected in the presence of a larger quantity of blurred spots in the micrographs of preform C, due to the large presence of nanoparticles under the surfaces analyzed. In preform C, nanoparticles in the range 17–160 nm were found with an average size of 75 nm in diameter while in preform D, nanoparticles of 14–120 nm with a mean diameter size of 50 nm were observed. Moreover, the concentration also affects the distance between the particles. In preform C some particles tend to be very close to each other, creating some agglomerates up to 240 nm (Fig. S3c), while in preform D, the nanoparticles are isolated and separated from each other by ~ 300 nm up to more than 2 microns. These microstructural changes associated to the reduction of the concentration from 0.01 to 0.005 M slightly increases the transparency of the core of the preform (Fig. S2).

Blanc et al.^32^ found experimentally that only concentrations of the soaking solution equal or higher than 0.1 M led to the formation of Ca-based nanoparticles in the core of silica-based preforms, with an average size of 250 nm. In these samples, phosphorus and germanium concentrations were around 1 mol.% and 2 mol.%, respectively. However, in the current work, much smaller nanoparticles are observed even for concentrations as low as 0.005 M, which demonstrates that experimental conditions in this type of silicate systems strongly condition the phase separation and growth of Ca-based nanoparticles. It is difficult to compare both works due to the strong influence of the different parameters involved in the experimental process^32^, but the results shown in our work might be explained by a larger quantity of Ge and P in the glass composition, which are modifying the phase equilibrium of SiO_2_-CaO, allowing the nucleation and growth of Ca-based nanoparticles under the experimental conditions explained in "Materials and methods"^28^. Due to the small size of the nucleated nanoparticles, their composition in preform C and D could not be extracted by EDX analyses, but based on the information obtained for preforms A and B and previous works, it is assumed that they are also Ca-enriched^28,32^. However, an EDX analysis of the whole core of the preforms was performed (Table 2) and confirmed that a silica-based glass of ~ 93 mol.% of SiO_2_ is formed, independently of the experimental conditions.

Therefore, these findings demonstrate the great influence of the studied variables involved in preform fabrication process such as vitrification temperature and concentration of soaking solutions, on the growth of Ca-based nanoparticles in the core of preforms. On the one hand, the reduction of vitrification temperature favors the nucleation of smaller Ca-based particles at a lower density since nucleation and growth time are decreased. On the other hand, by adjusting properly the concentration, it is possible to adapt the final nanometric size of the nanoparticles, which might be also promising for applications in which the encapsulation of rare-earth ions is involved.

### Tailoring nanoparticle features in Ca-based nanoparticles doped silica fibers: microstructural characterization

**Figure 2:**
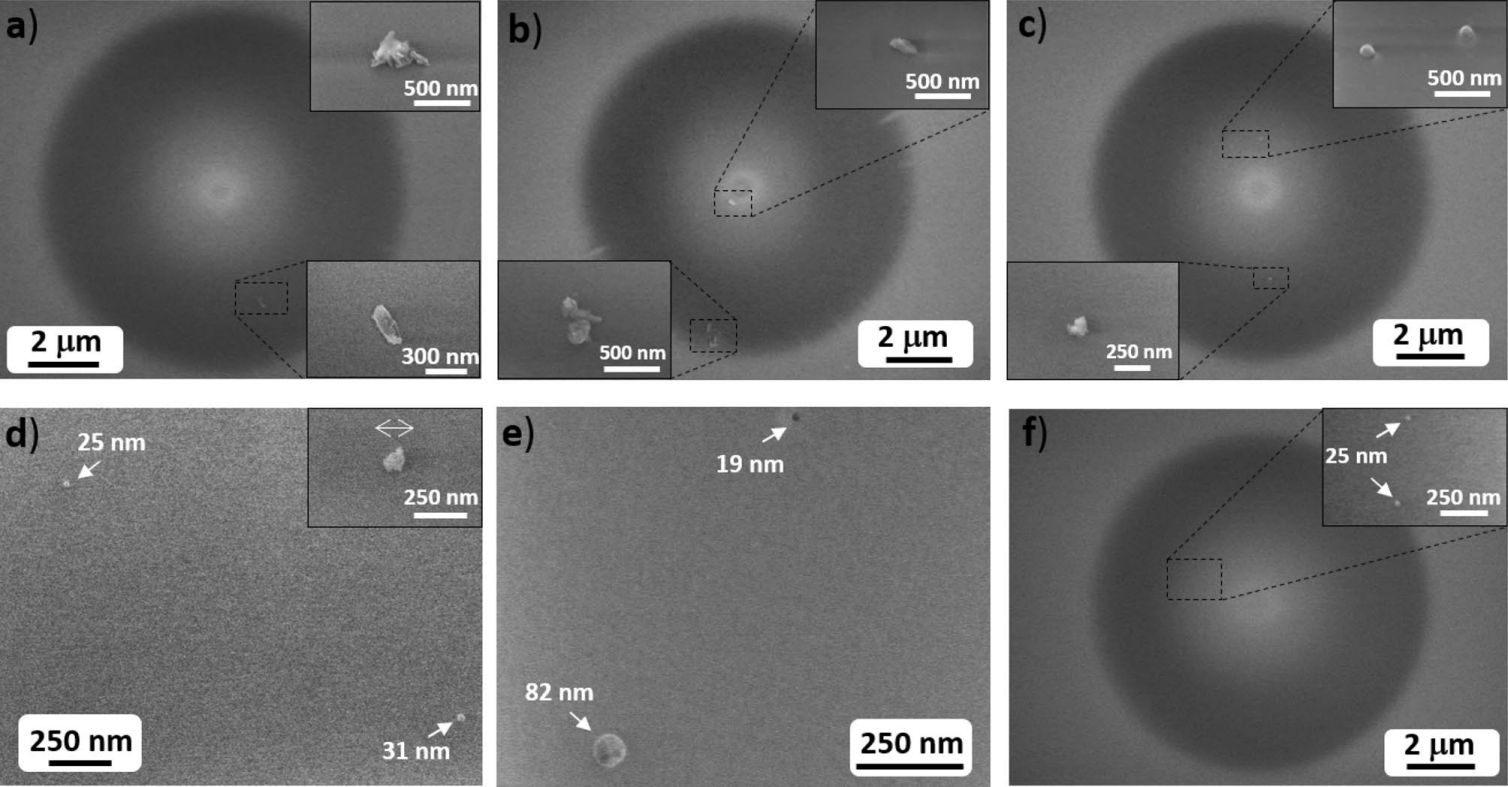
Representative SEM micrographs of fibers drawn from preform D at: (**a**) 1870 °C, (**b**) 1890 °C, (**c**) 1955 °C, (**d**) 2020 °C, (**e**) 2065 °C and (**f**) 2100 °C. The insets show in more detail the characteristic morphology and size of particles at the corresponding drawing temperature.

Since the aim of this work is to develop fibers combining enhanced Rayleigh scattering with the minimum optical losses possible, a low density of small nanoparticles, with a large inter-particle distance, in the fiber acting as DOFS is targeted. Therefore, the different fabricated fibers were made from preform D, based on the results discussed in section of the influence of preform fabrication conditions on nanoparticles features. The effect of the drawing temperature on the Ca-based nanoparticles present in the fibers was studied by analyzing SEM micrographs of ten sections for each sample and representative pictures are shown in Fig. 2. In general, it is observed that, as drawing temperature increases, from 1870 to 2200 °C, particle size considerably decreases and circularity of Ca-based particles increases. Density of particles remain very low for all fibers, decreasing as drawing temperature increases, and no more than five particles are generally observed in the surfaces analyzed. Moreover, in some surfaces no Ca-based particles were found, especially for high drawing temperatures, > 2100 °C. In the temperature range considered, the morphology and size evolution of Ca-based nanoparticles can be classified in three intervals well differentiated: (1) large particles of 214–500 nm in length in the interval 1870 °C ≤ T ≤ 1890 °C; (2) particles of 19–147 nm in diameter in the interval 1955 °C ≤ T ≤ 2065 °C and; (3) particles smaller than 25 nm at 2100 °C ≤ T ≤ 2200 °C. For 1870 °C ≤ T < 1890 °C, most of Ca-based particles have irregular elongated shapes (Fig. 2a,b and S4) despite the fact that they were spherical in the preforms (Fig. 1) although some spherical ones of 77–170 nm were also found (Fig. S4). Ca-based particles increase their size from approximately 50 nm, mean diameter size of nanoparticles in the corresponding preform, up to 234–500 nm (Fig. 2a and Fig. S4) and 214–396 nm (Fig. 2b and Fig. S4) in length in the fibers drawn at 1870 °C and 1890 °C, respectively. From 1955 °C, as temperature increases, Ca-based particles start to present spherical shapes with larger circularity and smaller sizes up to 115 nm (right-top inset of Fig. 2c). At this temperature, the average diameter size of the particles is 130 nm while no particle larger than 147 nm was found (left-bottom inset of Fig. 2c). Fibers drawn at 2020–2065 °C show highly spherical nanoparticles with diameters generally below 100 nm and a mean size of 83 nm and 70 nm, respectively. At temperatures above 2100 °C, most of Ca-based nanoparticles are probably dissolved since several analyzed surfaces did not show presence of any nanoparticle in the core of the fiber and only some nanoparticles up to 25 nm were found (inset Fig. 2f).

The observed behavior as a function of the drawing temperature allows attaining information about the kinetics of the system and suggests that the Ca-based nanoparticles presented in the core of the preform are dissolved during the drawing process. This is in accordance with the phase diagram of SiO_2_–CaO^34^. However, a subsequent nucleation occurs again in the core of the fiber, following a temperature dependent process. At lower drawing temperatures, larger particles were observed, which indicates that they are energetically more stable than smaller ones. An Ostwald ripening mechanism of nucleation and growth might be involved in this case. At higher temperatures, the observed behavior might be due to the higher solubility of the Ca-based particles in the silica-based glass, which causes that less amount of solute is available for the nucleation of new particles and therefore the growth is stopped. As a consequence, the number of nucleation centers decrease as well as the size, attaining smaller nanoparticles in a very low density, as it was previously discussed for temperatures above 2100 °C (Fig. 2f).

It is worth mentioning that Ca-based particles observed are randomly distributed in the core of the fibers, both in the inner and the outer parts (as depicted in Fig. 2a–c,f) and no ring pattern is followed, as occurred in Mg-based nanoparticles doped fibers^17,18^. Moreover, some particles were found outside of the core of the fiber but in much lesser extent. However, based on recent works, this is not a major drawback for enhancing light scattering in the fibers^35^. On the other hand, it has been observed in the cross-section SEM micrographs (Fig. 2) that the diameter of particles and their circularity is affected by drawing temperature. Moreover, in recent works^26^, it has been reported that particles within the core of the fiber are elongated along the drawing direction during the drawing process due to a flow-induced deformation. Thus, the morphology and size of nanoparticles in the fiber core is highly sensitive to drawing process in the different spatial directions. It is important to point out that such low concentration of nanoparticles and with large inter-particle distance, reported generally for all fibers of this work is a requirement to minimize optical losses associated with Rayleigh scattering. Future studies with techniques such as FIB-SEM^26^ will allow investigating larger volumes, attaining a larger statistics as well as information about the effect of drawing along that direction. Independently of the drawing temperature, the composition of the core of all fibers shows high homogeneity, with a content of GeO_2_ of 5.15–5.92 mol.% and P_2_O_5_ of 0.67–1.03 mol.%, as revealed by the EDX analysis carried out.

Blanc et al.^32^ found that Ca-based nanoparticles with a mean size of about 100 nm are presented in fibers drawn at temperatures higher than 2000 °C and made from a preform prepared by soaking the silica soot in a solution of 0.1 M. In our work, at drawing temperatures higher than 2000 °C, the samples doped with a solution 0.005 M show particles with a mean size of 25–83 nm. Obtaining smaller nanoparticles at lower concentrations is potentially important for applications of long-range DOFS or fiber lasers and amplifiers, since optical losses derived from Rayleigh scattering will be lessen.

These results demonstrate that the nucleation and growth of Ca-based particles in the core of silica-based optical fibers might be controlled by means of drawing temperature, with constant preform feed and drawing speed, which allows the tailoring of both particle size and distribution. It is worth noticing that these findings, previously discussed, may be applied to other nanoparticles doped silica fibers, allowing their tailored incorporation in fiber core for future applications.

### Influence of nanoparticle features on Rayleigh scattering enhancement: Optical backscatter reflectometry measurements

**Figure 3:**
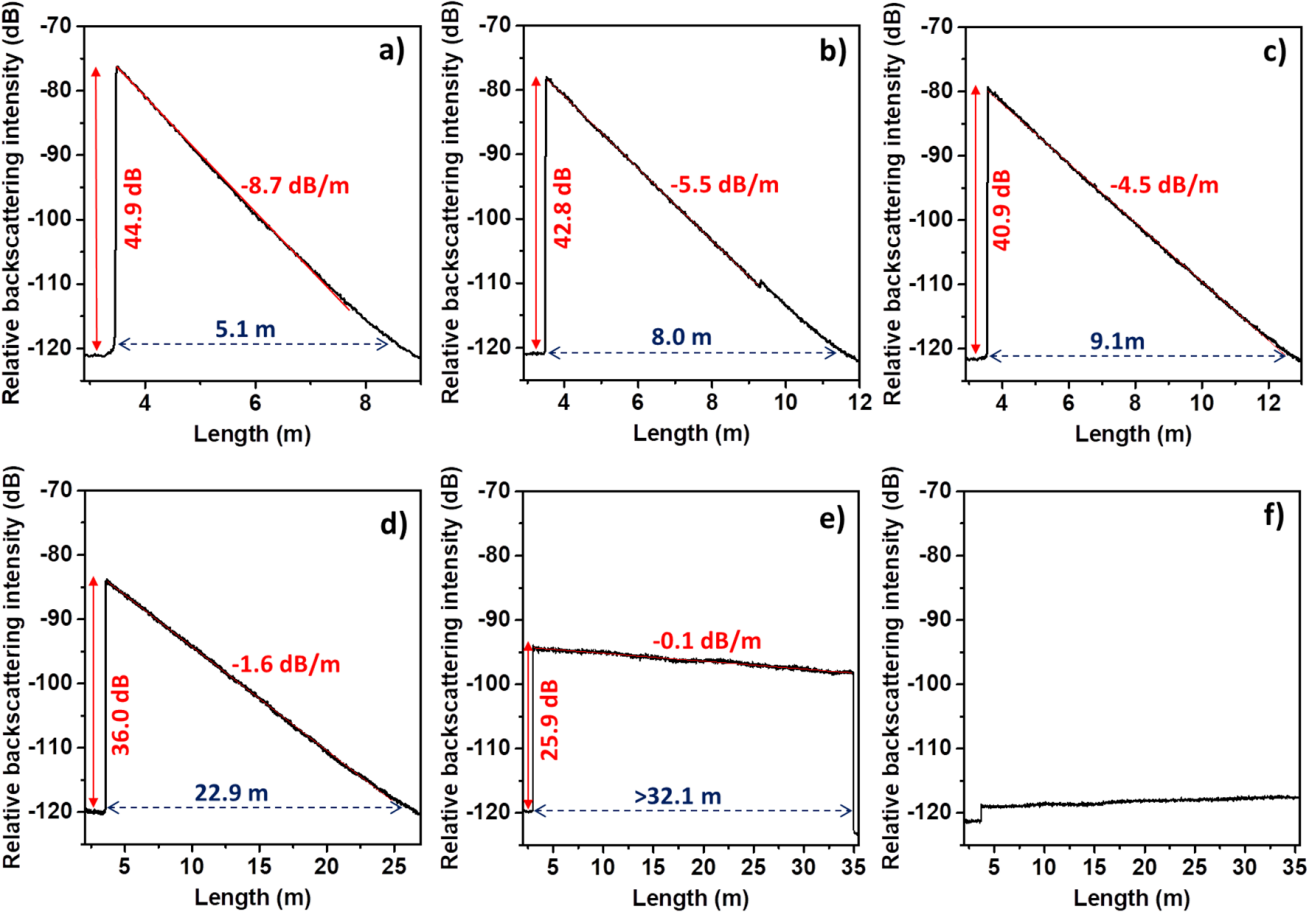
Backscattered intensity *vs* fiber length for Ca-based nanoparticles doped fibers drawn at different temperatures: (**a**) 1870 °C, (**b**) 1890 °C, (**c**) 1955 °C, (**d**) 2020 °C, (**e**) 2065 °C and (**f**) 2100 °C. No backscattering enhancement was observed in fibers drawn at temperatures higher than 2100 °C.

Figure 3 shows the enhanced Rayleigh backscattered intensity along the fiber length for the Ca-based nanoparticles doped fibers drawn at different temperatures: 1870 °C (Fig. 3a), 1890 °C (Fig. 3b), 1955 °C (Fig. 3c), 2020 °C (Fig. 3d), 2065 °C (Fig. 3e) and 2100 °C (Fig. 3f). The enhancement of Rayleigh backscattered intensity is determined by the difference between the measured amplitude signal before the splice point, with the SMF-28 fiber taken as reference, and after, and it is indicated with an arrow in Fig. 3. It can be seen that all fibers drawn at temperatures below 2065 °C present a variable enhancement of Rayleigh backscattering signal, in the range 25.9–44.9 dB. This enhancement in Rayleigh backscattering is induced by the Ca-based nanoparticles grown in situ inside the core of the fiber that act as Rayleigh scattering centers. Consequently, these scattering centers induce simultaneously an optical attenuation, whose average values are in the range 0.1–8.7 dB/m (considering forward and backward scattering). Fibers drawn at 1870–2065 °C (Fig. 3a–e) show a sawtooth shape, in which backscattering seems to be quite homogeneous along the fiber length and therefore attenuation could be estimated by a linear regression of the part marked by a red line. As temperature increases, scattering level decreases and thus optical attenuation is also reduced, which can be appreciated from the changes in the slope of backscattering intensity patterns. At 2020 °C (Fig. 3d), the intensity drops at a rate of 1.6 dB/m along the fiber length, which allows that the enhanced signal, 36.0 dB, is propagated around 22.9 m before being extinguished. At 2065 °C (Fig. 3e), the signal is not extinguished after propagating ~ 32.1 m, being limited by the length of the fiber. The enhanced signal of 25.9 dB has a two-way attenuation as low as 0.1 dB/m, which will allow the distributed sensing over long distances, up to 259 m. Fiber drawn at 2100 °C shows very low backscattering enhancement, almost at the noise level, while no backscattering enhancement was observed in fibers drawn at temperatures higher than 2100 °C.

**Figure 4:**
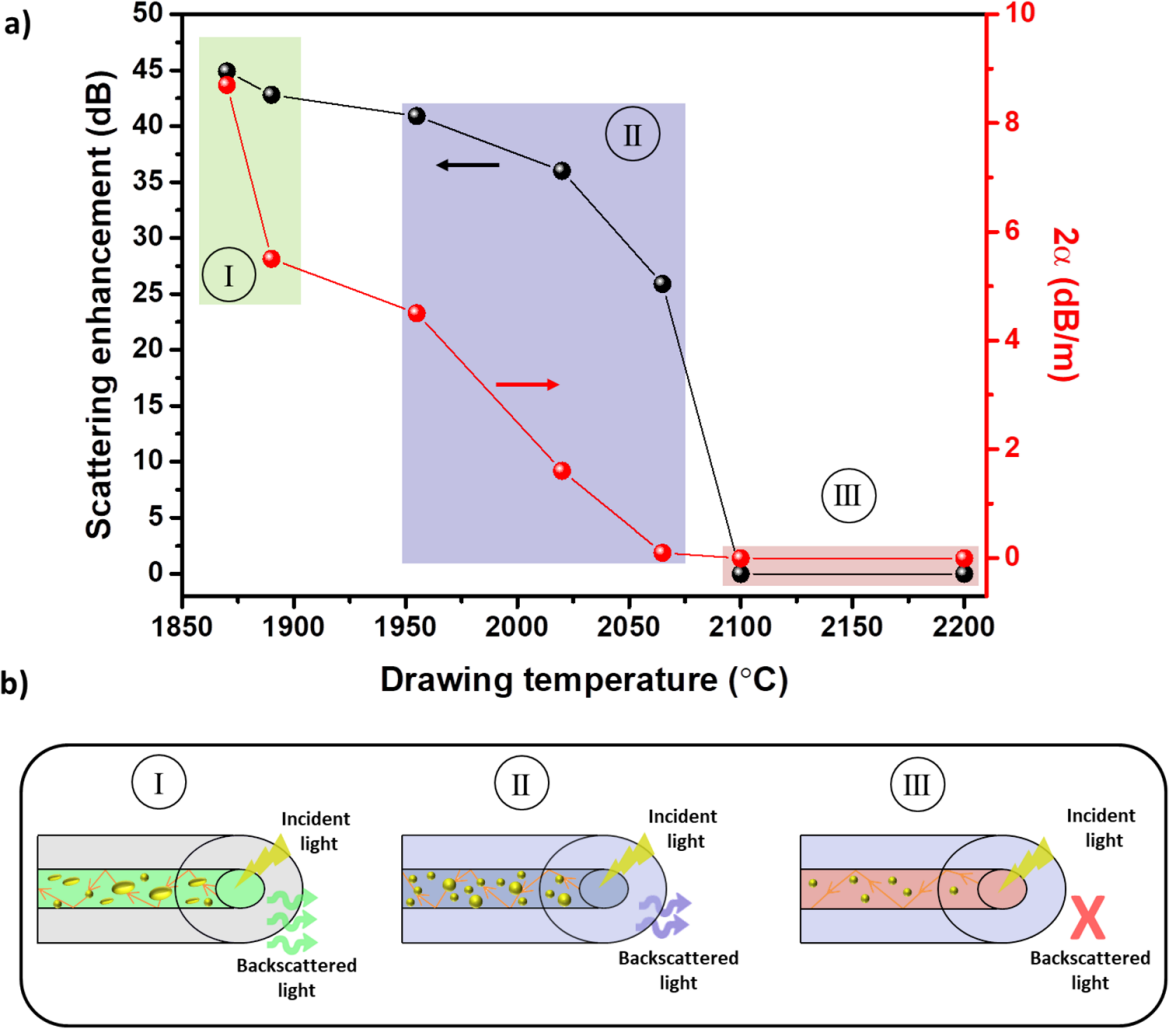
(**a**) Scattering enhancement (left axis) and two-way attenuation, 2α, (right axis) *versus* drawing temperature for Ca-based nanoparticles doped silica fiber. (**b**) Schematic model showing the influence of drawing temperature on the morphology and size of Ca-based nanoparticles inside the fiber core and their correlation with the scattering enhancement.

The correlation between backscattering enhancement and the two-way attenuation *versus* the drawing temperature is plotted in Fig. 4a and it can be explained as follows. At drawing temperatures below 1890 °C, most of the particles observed are elongated and large, up to 500 nm in length. Therefore, the scattered light is higher and consequently the optical loss also increases. For drawing temperatures between 1955–2065 °C, spherical particles smaller than 147 nm are found, with a diameter size that decreases progressively as temperature increases, as it was previously discussed in the section of microstructural characterization of fibers. Thus, the backscattering enhancement and the propagation loss are reduced from 40.9 and 4.5 dB/m, respectively, to 25.9 dB and 0.1 dB/m, approximately. Finally, at temperatures higher than 2100 °C, the density of nanoparticles found was very low and only some nanoparticles of ~ 25 nm were observed, as revealed SEM observations. Therefore, they do not enhance properly the Rayleigh backscattered light in the fibers. The schematic model plotted in Fig. 4b represents the explained behavior.

Based on Eqs. (1) and (2) (see Materials and methods), $\sigma$ and therefore $\alpha$ are proportional to Ø^6^ and thus the diameter of nanoparticles in the core of the fibers is a critical parameter to control during the fiber drawing, in order to attain an accurate enhancement in the scattering of the light with relatively low optical losses. Since its dependence is of a sixth power, having nanoparticles with a size larger than 100 nm will generally induce losses of hundreds of dBs^23^. However, the linear dependence of attenuation with N (Eq. 1) makes particle density another important parameter to be taken into consideration. In this work, it is demonstrated that despite having large Ca-based nanoparticles, even larger than 100 nm, it is possible to induce a high scattering enhancement, up to 44.9 dB, with relatively low optical losses, < 9 dB/m (two-way), by controlling their density in the core of the fiber.

With the aim of verifying the impact of the concentration on the scattering enhancement in Ca-based nanoparticles doped silica fibers, a fiber was drawn from preform C at 2020 °C. Its optical and microstructural characterization is shown in Fig. S5. This temperature was chosen because of the good trade-off between scattering enhancement and optical loss that fiber drawn from preform D presents (Fig. 3d). Figure S5a shows that the enhancement of the Rayleigh backscattered intensity along the fiber length is 53.1 dB, compared with the SMF-28 fiber taken as reference. This fiber displays the largest backscattering reported so far but optical attenuation is still high for long-range distributed sensing, > 100 dB/m. In contrast, the fiber drawn at the same temperature from preform D exhibits values of 36.0 dB and 1.6 dB/m, respectively (Fig. 3d). A representative SEM micrograph of this fiber is shown in Fig. S5b, in which a higher density of larger particles is clearly observed. Ca-based nanoparticles size ranges from 45 to 287 nm in this type of fiber, while the average diameter size found was 171 nm. However, the corresponding fiber drawn at 2020 °C (preform prepared with the solution 0.005 M) showed much isolated nanoparticles with an average diameter of 83 nm. From these results, it can be confirmed that the soaking concentration for preform fabrication plays also a very critical role, since double the concentration from 0.005 M (Fig. 3d) to 0.01 M (Fig. S5a) causes an increase of the Rayleigh backscattered intensity by a factor ~ 1.5, while attenuation is increased by a factor ~ 100. As it was discussed in section of preform fabrication, Ca-based nanoparticles in the preform C tend to be closer from each other than in preform D, and even forming some agglomerates. This fact favors that new calcium-based nuclei are also closer in the melted silica and when mass transport occurs during drawing process, higher calcium content is available to nucleate and grow; consequently, the particles also develop a larger size in a higher density, as it is observed.

In the literature, so far, the in-depth studies of nanoparticle-doped fibers for distributed sensing applications are based on MgO-based nanoparticles co-doped with erbium, with different sizes and configurations. Best results have been reported when MgO-based nanoparticles of Ø < 100 nm are distributed in the inner core of the fibers, which leads to an increase of the Rayleigh scattering of 32.3–45.2 dB with a two-way attenuation of 27.8–33.1 dB/m^19–22^. On the other hand, backscattering enhancement values of 46.1–47.5 dB are attained when nanoparticles of 20–160 nm are placed in a ring around the core and around 300 dB/m of attenuation^17,18^. Very recently, MgO-based nanoparticles fibers have been optimized even more, allowing an increase of the Rayleigh scattering up to 48.9 dB with a two-way attenuation of 14.3 dB/m^24^. The low concentration and high dispersion of the Ca-based nanoparticles in the core of the fibers developed in our work, allow having a superior performance, with the lowest two-way attenuation (Figs. 3, 4) reported so far in the state-of-the-art for Rayleigh scattering enhanced nanoparticles-doped silica fibers, which permits longer sensing distances from 5 m up to 259 m, while Rayleigh scattering enhancement reaches values up to 44.9 dB.

The works of Blanc et al., along with the results reported in the current work show the suitability and wide range of opportunities that nanoparticles doped optical fibers offer for DOFS, since fabrication parameters, such as drawing temperature can control particle size, as well as their arrangement in the fiber core, allowing adapting their features to the desired applications. Moreover, nanoparticles doped optical fibers have some additional advantages related to other approaches followed in literature so far, such as the UV exposure of SMF^9,13,15^, and the inscription of nanogratings by a femtosecond laser^16^. Regarding the last one, the inscription process is too slow to be implemented in long length sensing systems, since no Rayleigh scattering enhancement is found for laser writing speeds exceeding 2 mm/s^16^. On the other hand, nanoparticles-doped fibers may be manipulated as a standard SMF fiber and their protective jacket might be preserved, ensuring a good mechanical reliability, which is a key criteria for many sensing applications, while for UV exposure and the nanogratings inscription, it must be removed^12^.

### Performance of Ca-based nanoparticles doped silica fibers in optical communication wavelength bands: Attenuation measurements

**Figure 5:**
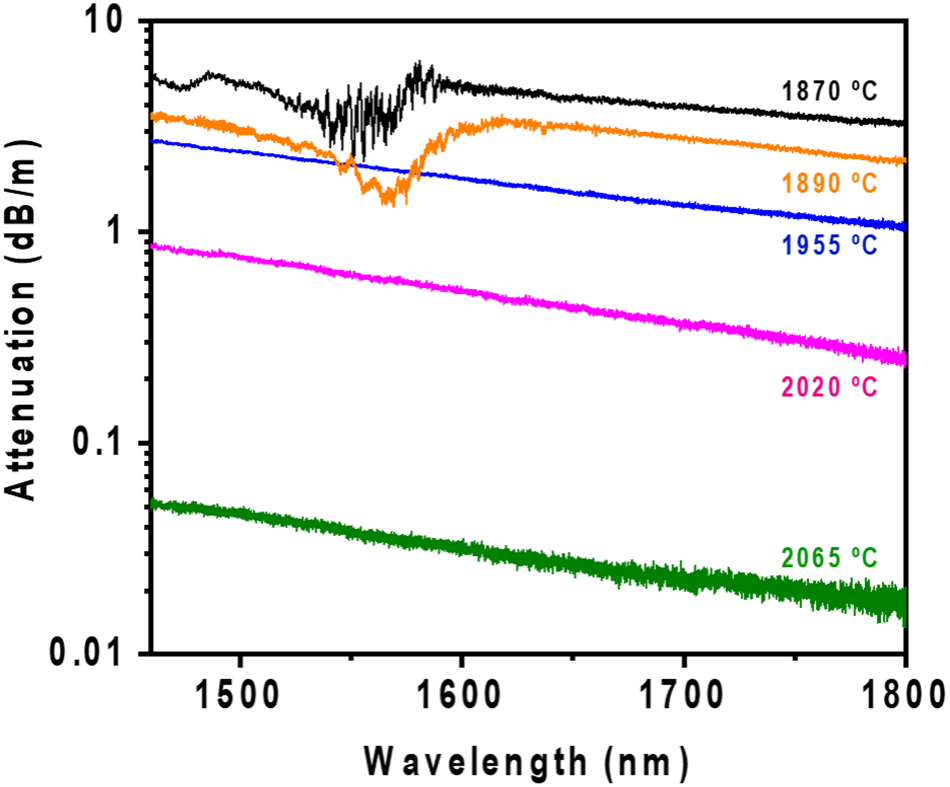
Fiber attenuation *vs* wavelength (nm) for the enhanced scattering fibers drawn from preform D at temperatures in the range 1870–2065 °C. Fibers drawn at temperatures above 2065 °C are not considered since no backscattering enhancement was registered. Linear fits for the range 1620–1800 nm and 1500–1800 nm, are included in the plot for fibers drawn at 1870–1890 °C and 1955–2065 °C, respectively.

To study the performance of the enhanced scattering fibers over a wider wavelength range (1450–1800 nm) and measure their attenuation values more precisely, the standard cutback method was used. The results are plotted in Fig. 5.

In Fig. 5, it can be seen that as drawing temperature of Ca-based nanoparticles doped fibers increases, attenuation decreases for the whole wavelength range studied. These results agree well with the ones previously discussed, since the higher is the drawing temperature, the smaller is the size of the nanoparticles in the core and thus a lower scattering and attenuation are expected. At drawing temperatures below 1900 °C, most of the particles observed have elongated and irregular shapes as well as large size, > 200 nm in length, although some spherical of ~ 100 nm were also found, but in lesser extent. These features make that the scattering of light follows a more complex behavior at wavelengths shorter than 1600 nm, having a non-linear trend, due to the contribution of the irregular and large particles, while the spherical smallest ones are less perceptible for the incident light^36^. At longer wavelengths, the Rayleigh scattering contribution starts to be more considerable and therefore the attenuation curve is softened by a decreasing linear trend proportional to λ^−4^ (Eq. 2), as it has been also observed for MgO nanoparticle doped fibers^24^. However, from 1955 °C, smaller nanoparticles, < 147 nm, with a spherical shape were found as revealed SEM micrographs showed in section of microstructural characterization of fibers and therefore Rayleigh scattering is more predominant for the rest of the fibers. This major source of scattering is revealed by the decreasing tendency proportional to λ^−4^ followed by all attenuation curves in all the wavelength range considered (Fig. 5). The low losses of these fibers in the near infrared along with the tunable scattering enhancement suggest that Ca-based nanoparticles doped silica fibers might be considered as potential candidates for DOFS applications even beyond the C-band. Future works will investigate the application of this type of fibers in the L-band.

Most of the particles observed in fibers drawn at temperatures below 1900 °C are elongated and larger than 200 nm and although some spherical were observed, the statistic is low. Therefore Eq. (2) for the Rayleigh scattering regime cannot be applied. However, considering that this regime might be applied for nanoparticles with Ø < λ/10 and by using Eqs. (1)–(5), *N* might me estimated for fibers drawn at 1955–2065 °C, making the following assumptions. Owing to the exact composition of the nanoparticles for each fiber is not known, refractive index is considered as a constant for all, which allows us to make a relative comparison and discuss the effect of concentration and diameter size. The refractive index of SiO_2_ is 1.45, for CaO is 1.84^37^ and 1.53 for CaO–4SiO_2_ glasses (CaO content of ~ 19 wt.%)^38^ Based on the EDX analysis presented in section of microstructural characterization of fibers, a n_np_ ~ 1.7 is assumed for the estimation, considering that CaO content is ~ 33 wt.% and GeO_2_ and P_2_O_5_ also increase the refractive index of silica. Assuming n_med_ = 1.45, the mean particle diameter attained from SEM observations is ∅ = 130 nm, 83 nm, 70 nm and α value at 1550 nm is 2 dB/m, 0.6 dB/m and 0.04 dB/m, for fibers drawn at 1955 °C, 2020 °C, 2065 °C, respectively. The particle density estimated is 9.1 × 10^17^, 4.1 × 10^18^ and 6.6 × 10^17^ particles/m^3^, respectively, with an associated uncertainty of ~ 1.0 × 10^17^. It is important to emphasize that the above discussion is an estimation, and not a precise calculation, since consider refractive index homogeneous for all nanoparticles and independent of the drawing process, with the aim of elucidating the possible renucleation mechanism involved. Based on these estimations, it can be suggested that as drawing temperature increases, nucleation of Ca-based particles increases progressively while their size decreases, reaching a maximum at 2020 °C and afterwards decreasing sharply for temperatures above 2100 °C, as it was previously discussed. Furthermore, a similar estimation might be done in the fiber drawn at 2020 °C from preform C, considering ∅ = 171 nm and α ≈ 78 dB/m (from OBR measurements). In this case, a particle density of 6.6 × 10^18^ particles/m^3^ is calculated in the fiber. Thus, from these estimations, it can be concluded that doubling the concentration of the solution during preform preparation also double approximately the density of particles in the fiber as well as their mean diameter size, which leads to an increase of the optical losses by a factor ~ 100.

It is worth mentioning that these findings evince that backscattering enhancement and optical attenuation in nanoparticles doped silica optical fibers is extremely sensitive to experimental conditions and might be easily tailored through careful control of preform and fiber fabrication conditions, as it has been presented in this work. These results pave the way for future works in the field of nanoparticles-doped optical fibers for distributed sensing applications.

## Conclusions

Low-loss Ca-based nanoparticles doped silica fibers with tunable Rayleigh scattering have been fabricated by MCVD method to be used in long-range distributed sensing applications. In this work, it has been demonstrated that experimental conditions during preform fabrication process, such as concentration of solution and vitrification temperature, have a direct impact on the features of the Ca-based nanoparticles in the silica fibers. Decreasing simultaneously vitrification temperature from 1950 to 1800 °C, and solution concentration from 0.1 to 0.005 M, allows decreasing the density and the size of Ca-based nanoparticles in the preforms core from several μm to less than 100 nm, attaining dispersed nanoparticles with a large inter-particle distance. During fiber drawing process, nanoparticles are dissolved and re-nucleation occurs as a function of drawing temperature, which allows the engineering of their features. As drawing temperature increases, in the range 1870–2200 °C, the Ca-based particles experience a considerable change in their morphology and size, becoming spherical and decreasing from 500 nm in length down to 25 nm in diameter. The low concentration and high dispersion of the nanoparticles in the fibers core allow having the lowest two-way attenuation reported so far for Rayleigh scattering enhanced nanoparticles-doped silica fibers, which permits longer sensing distances from 5 m up to more than 200 m. These two-way losses are comprised between 0.1 and 8.7 dB/m, with an associated Rayleigh scattering enhancement compared to SMF-28 fiber that can be tuned in the range 25.9–44.9 dB, respectively. These results illustrate the potential of Ca-based nanoparticles doped optical fibers for long-distance distributed sensing and show the outstanding possibilities that nanoparticles-doped optical fibers offer for this kind of applications. It should be noticed that these findings can be considered as a reference for future works in this research topic, since the tailoring of DOFS may be attained by appropriate controlling of MCVD and fiber drawing conditions.

## Supplementary Information

Supplementary Information

## Data Availability

The raw/processed data required to reproduce these findings cannot be shared at this time as the data also forms part of an ongoing study.

## References

[1] Dakin JP (1993). Distributed optical fiber sensors. Proc. SPIE 10266 Fiber Opt. Sens. A Crit. Rev..

[2] Giallorenzi T, Bucaro JA, Dandridge A, Sigel GH, Cole JH (1982). Optical fiber sensor technology. IEEE Trans. Microw. Theory Tech..

[3] Tosi, D., Molardi, C., Blanc, W., Marques, C. & Sales, S. Multiplexing techniques and applications in fiber-optic spatially resolved sensing networks. *Opt. Sensors Sens. Congr. (ES, FTS, HISE, Sensors), OSA Tech. Dig. (Optical Soc. Am. 2019), Pap. STh4A.2* STh4A.2. 10.1364/sensors.2019.sth4a.2.

[4] Ding Z (2018). Distributed optical fiber sensors based on optical frequency domain reflectometry: A review. Sensors (Switzerland).

[5] Palmieri L, Schenato L (2013). Distributed temperature sensing based on Rayleigh scattering. Open Opt. J..

[6] Lu P (2019). Distributed optical fiber sensing: Review and perspective. Appl. Phys. Rev..

[7] Xu C, Khodaei ZS (2020). Shape sensing with Rayleigh backscattering fibre optic sensor. Sensors.

[8] Bado MF, Casas JR, Barrias A (2018). Performance of Rayleigh-based distributed optical fiber sensors bonded to reinforcing bars in bending. Sensors.

[9] Loranger S, Gagné M, Lambin-Iezzi V, Kashyap R (2015). Rayleigh scatter based order of magnitude increase in distributed temperature and strain sensing by simple UV exposure of optical fibre. Sci. Rep..

[10] Bao X, Chen L (2012). Recent progress in distributed fiber optic sensors. Sensors.

[11] Tosi D (2020). Performance analysis of scattering-level multiplexing (SLMux) in distributed fiber-optic backscatter reflectometry physical sensors. Sensors.

[12] Tosi D, Molardi C, Sypabekova M, Blanc W (2020). Enhanced backscattering optical fiber distributed sensors: Tutorial and review. IEEE Sens. J..

[13] Parent F (2017). Enhancement of accuracy in shape sensing of surgical needles using optical frequency domain reflectometry in optical fibers. Biomed. Opt. Express.

[14] Jin J, Zhang H, Liu J, Li Y (2016). Distributed temperature sensing based on Rayleigh scattering in irradiated optical fiber. IEEE Sens. J..

[15] Parent F (2019). Intra-arterial image guidance with optical frequency domain reflectometry shape sensing. IEEE Trans. Med. Imaging.

[16] Yan A (2017). Distributed optical fiber sensors with ultrafast laser enhanced Rayleigh backscattering profiles for real-time monitoring of solid oxide fuel cell operations. Sci. Rep..

[17] Ayupova T (2020). Fiber optic refractive index distributed multi-sensors by scattering-level multiplexing with MgO nanoparticle-doped fibers. IEEE Sens. J..

[18] Korganbayev S (2019). Refractive index sensor by interrogation of etched MgO nanoparticle-doped optical fiber signature. IEEE Photonics Technol. Lett..

[19] Beisenova A (2019). Distributed fiber optics 3D shape sensing by means of high scattering NP-doped fibers simultaneous spatial multiplexing. Opt. Express.

[20] Beisenova A (2019). Simultaneous distributed sensing on multiple MgO-doped high scattering fibers by means of scattering-level multiplexing. J. Light. Technol..

[21] Beisenova A (2019). Multi-fiber distributed thermal profiling of minimally invasive thermal ablation with scattering-level multiplexing in MgO-doped fibers. Biomed. Opt. Express.

[22] Sypabekova M (2018). Fiber optic refractive index sensors through spectral detection of Rayleigh backscattering in a chemically etched MgO-based nanoparticle-doped fiber. Opt. Lett..

[23] Blanc W (2011). Fabrication of rare earth-doped transparent glass ceramic optical fibers by modified chemical vapor deposition. J. Am. Ceram. Soc..

[24] Tosi D, Molardi C, Blanc W (2021). Rayleigh scattering characterization of a low-loss MgO-based nanoparticle-doped optical fiber for distributed sensing. Opt. Laser Technol..

[25] Molardi C (2019). Fiber Bragg Grating (FBG) sensors in a high-scattering optical fiber doped with MgO nanoparticles for polarization-dependent temperature sensing. Appl. Sci..

[26] Vermillac M (2017). Fiber-draw-induced elongation and break-up of particles inside the core of a silica-based optical fiber. J. Am. Ceram. Soc..

[27] Vermillac M (2019). On the morphologies of oxides particles in optical fibers: Effect of the drawing tension and composition. Opt. Mater. (Amst).

[28] Blanc W, Dussardier B, Paul MC (2009). Er-doped oxide nanoparticles in silica-based optical fibers. Glas Technol. Eur. J. Glas. Sci. Technol. A.

[29] Blanc W, Mauroy V, Dussardier B (2012). Erbium-doped nanoparticles in silica-based optical fibres. Int. J. Nanotechnol..

[30] Cox AJ, DeWeerd AJ, Linden J (2002). An experiment to measure Mie and Rayleigh total scattering cross sections. Am. J. Phys..

[31] Marcuse D (1977). Loss analysis of single-mode fiber splices. Bell Syst. Tech. J..

[32] Blanc W (2009). Erbium emission properties in nanostructured fibers. Appl. Opt..

[33] Blanc W, Guillermier C, Dussardier B (2012). Composition of nanoparticles in optical fibers by secondary ion mass spectrometry. Opt. Mater. Express.

[34] White JF, Lee J, Hessling O, Glaser B (2017). Reactions between liquid CaO–SiO2 slags and graphite substrates. Metall. Mater. Trans. B Process Metall. Mater. Process..

[35] Lanzarini-Lopes M (2019). Particle-modified polymeric cladding on glass optical fibers enhances radial light scattering. J. Opt. Soc. Am. B.

[36] Bohren CF, Huffman RD (1998). Absorption and scattering of light by small particles.

[37] Samsonov GV (1973). The Oxide Handbook.

[38] Hayashi T, Saito H (1980). Preparation of CaO–SiO_2_ glasses by the gel method. J. Mater. Sci..

