# Supplementary material for: Engineering nanoparticle features to tune Rayleigh scattering in nanoparticles-doped optical fibers
Source: Sci Rep. 2021 Apr 27;11:9116. doi: 10.1038/s41598-021-88572-2 (PMC8079377; doi:10.1038/s41598-021-88572-2)
Supplement: Supplementary file 1 — Supplementary Information [file 41598_2021_88572_MOESM1_ESM.docx]

**Supporting information**

Engineering Nanoparticle Features to Tune Rayleigh Scattering in Nanoparticles-doped Optical Fibers

*Victor Fuertes^*1^, Nicolas Grégoire^1^, Philippe Labranche^1^, Stéphane Gagnon^1^, Ruohui Wang^1^, Yannick Ledemi^1^, Sophie LaRochelle^1^, Y. Messaddeq^1^*

^1^Centre d’optique, Photonique et Laser, 2375 Rue de la Terrasse, Université Laval, Québec, (QC), G1 V 0A6, Canada

*Corresponding Author: [victor.fuertes-de-la-llave.1@ulaval.ca](mailto:victor.fuertes-de-la-llave.1@ulaval.ca)

**S1.** **Refractive index profiles of Ca-based nanoparticles doped preforms**

**Fig. S1**. Refractive index profiles of Ca-based nanoparticles doped preforms B-D. Refractive index contrast of preform A could not be evaluated because of the high density of particles.

**S2. Photograph of a section of preforms A-D showing the different core transparency**

**Fig. S2**. Photograph taken of a section of preforms A-D studied in this work, showing an increase of transparency of the core as soaking concentration and vitrification temperature decrease, that is, from preform A to preform D.

.

**S3. Additional SEM micrographs for preform C and D**


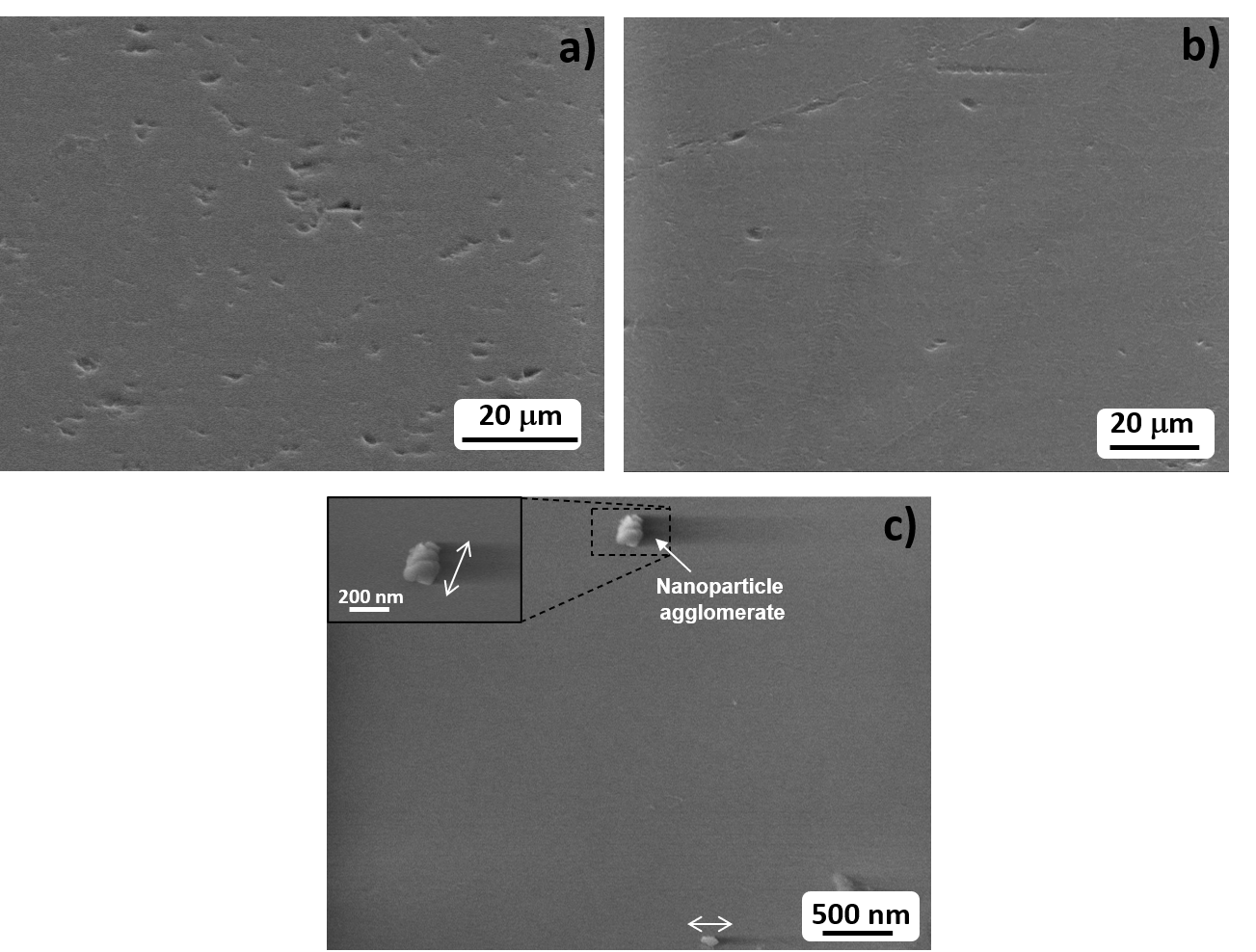


**Fig. S3**. SEM micrographs showing the core of: **a)** preform C and **b)** preform D, at low magnification, evincing the absence of micrometric particles; **c)** preform C, at higher magnification, highlighting the presence of some nanoparticle agglomerate. The observed holes of the surface are caused by polishing process.

**S4. SEM micrographs showing characteristics particles for fibers drawn at 1870 ≤T<** **1890 °C from preform D.**

**Fig. S4**. SEM micrographs showing characteristic morphology and size of the particles presented in fibers drawn from preform D at: **a,b)** 1870 °C and **c,d)** 1890 °C. In this range of temperatures elongated particles are predominant, generally with irregular shapes, although some particles with larger circularity are also found.

**S5. Characterization of fiber drawn at 2020 ºC from preform C**


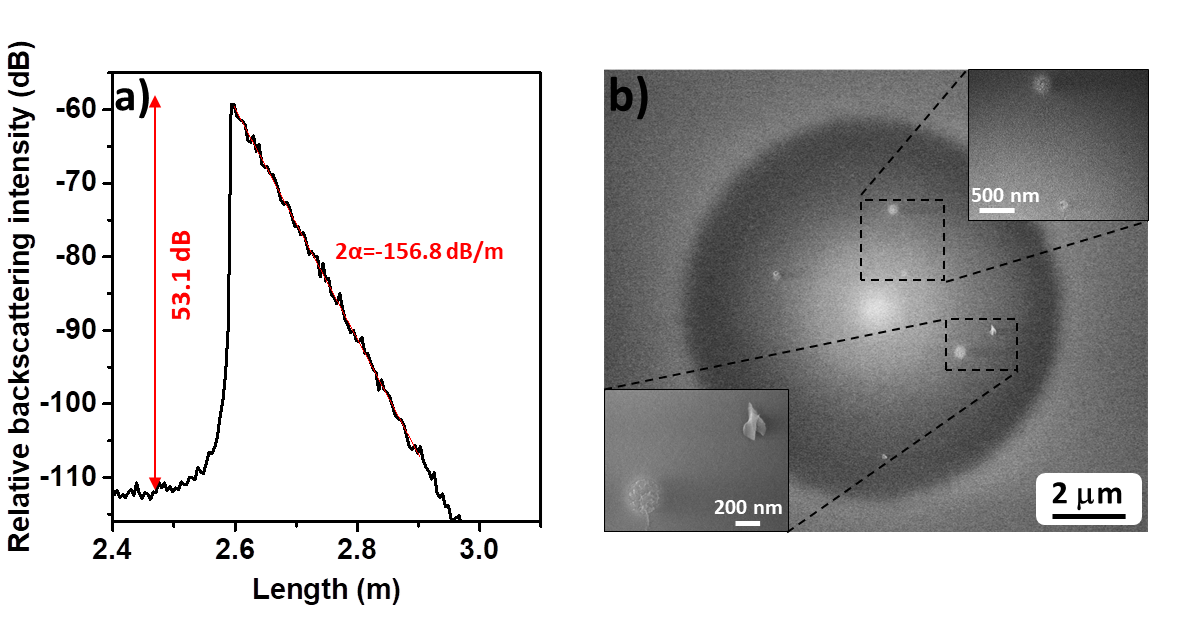


**Fig. S5**. **a)** Backscattered intensity *vs* fiber length for fiber drawn at 2020 °C from preform C. **b)** SEM micrograph of the core, showing a higher density of particles and bigger size than the corresponding ones in the fiber drawn at the same temperature from preform D. The characteristic morphology and size of the particles is shown in more detail in the enlargements of the marked areas.
